# Factors Associated With Endodontic Flare‐Ups: A Systematic Review and Meta‐Analysis

**DOI:** 10.1111/iej.70164

**Published:** 2026-04-23

**Authors:** Jun Ohshima, Masayoshi Morita, Yuzo Kawanishi, Shotaro Abe, Nobutake Tanaka, Tsuyoshi Shimaoka, Hazuki Maezono, Yoshifumi Kinomoto, Mikako Hayashi

**Affiliations:** ^1^ Department of Restorative Dentistry and Endodontology Graduate School of Dentistry, the University of Osaka Suita Osaka Japan; ^2^ Department of Endodontics Graduate School of Dentistry, Osaka Dental University Hirakata Osaka Japan

**Keywords:** endodontics, pain management, pain measurement, periapical periodontitis, postoperative complications, postoperative pain, treatment outcome

## Abstract

**Background:**

Endodontic flare‐ups, characterized by severe postoperative pain and/or swelling requiring unscheduled intervention, remain a clinically significant concern in root canal therapy. Identifying the factors associated with flare‐ups is important for anticipating and managing patient outcomes.

**Objectives:**

This systematic review and meta‐analysis aimed to identify and synthesize clinical risk factors associated with endodontic flare‐ups following root canal treatment.

**Methods:**

A comprehensive literature search was conducted in PubMed, Web of Science, and Scopus from database inception to September 2025. Eligible studies included observational or interventional studies evaluating the association between potential risk factors and flare‐up occurrence. Risk of bias was assessed using ROBINS‐I for non‐randomized studies and the Cochrane RoB 2 tool for randomized controlled trials. Certainty of evidence was evaluated using the GRADE approach.

**Results:**

A total of 15 eligible studies involving 24 320 cases met the inclusion criteria. The overall incidence of severe flare‐ups was 2.83%. Significant risk factors included female sex, mandibular tooth location, non‐vital pulp status, preoperative spontaneous pain, periapical lesions, multiple‐visit treatment, re‐treatment, and preoperative percussion pain. Among these, preoperative spontaneous pain (relative risk = 5.83) and percussion pain (relative risk = 3.45) demonstrated the strongest associations. By contrast, age, tooth type, number of root canals, instrumentation technique, and preoperative analgesic use were not significantly associated with flare‐up incidence.

**Conclusion:**

Several clinical factors appear to be associated with an increased risk of endodontic flare‐ups. Although the overall certainty of evidence is low, these findings may assist clinicians in identifying higher‐risk cases and counselling patients regarding potential postoperative outcomes. Further well‐designed prospective studies are needed to strengthen the evidence base.

**Trail Registration:**

PROSPERO Systematic review registration number: CRD420251025451

## 1 Introduction

Endodontic flare‐ups are acute exacerbations of periapical inflammation that occur unexpectedly within a few days after root canal treatment. The American Association of Endodontists defines a flare‐up as “an acute exacerbation of an asymptomatic pulpal and/or periradicular pathosis following the initiation or continuation of root canal treatment” (American Association of Endodontists n.d.). Flare‐ups are characterized by severe pain and/or swelling, which can increase patient anxiety and distress (Seltzer and Naidorf 2004a). Patients may perceive flare‐ups as treatment‐related, potentially undermining trust in the dentist (Van Wijk and Hoogstraten 2006). Despite recent advances in endodontic techniques and materials, flare‐ups can still occur even when root canal treatment is performed carefully and appropriately, often resulting in emergency visits after the procedure. In addition to compromising patient comfort and satisfaction, flare‐ups increase the need for emergency care, disrupt clinical workflow, and prolong overall treatment time. To improve clinical outcomes and patient care in root canal treatment, the factors contributing to these flare‐ups must be better understood.

The reported incidence of endodontic flare‐ups varies widely in the literature, ranging from 0.39% (Iqbal et al. 2009) to 38% (Almudahki et al. 2022), while evidence regarding contributing factors remains limited (Walton 2002). This considerable variation is largely attributable to differences in flare‐up definitions and, to some extent, study design. Some investigations have applied relatively broad criteria, such as any increase in postoperative pain on a visual analogue scale (VAS) or the use of analgesics, whereas others have adopted stricter definitions requiring pain and/or swelling severe enough to necessitate an unscheduled emergency visit and additional intervention. Because pain intensity is inherently subjective and mild postoperative discomfort is common, distinguishing a true flare‐up from expected postoperative pain remains challenging (Pak and White 2011). Moreover, when flare‐ups are defined strictly, their relatively low frequency makes adequately powered prospective studies difficult; conversely, broader definitions used to increase event counts tend to yield higher reported incidence rates.

The aetiology of endodontic flare‐ups is multifactorial and can be conceptualized as an acute exacerbation of periapical inflammation that occurs when pre‐existing infection and host susceptibility intersect with treatment‐related insults (Seltzer and Naidorf 2004a). From a microbiological perspective, persistent intracanal infection and a high microbial load may prime periapical tissues; subsequent instrumentation or irrigation can disrupt biofilms and release microbial components, amplifying local inflammatory signalling (Trope 1991; Siqueira 2003). Mechanically, overinstrumentation, apical extrusion of infected debris, or overfilling may introduce bacteria and irritants into periapical tissues, increasing tissue pressure and provoking an acute inflammatory response that clinically manifests as severe pain and/or swelling (Seltzer and Naidorf 2004a). Chemical irritation from irrigants or intracanal medicaments may further aggravate inflamed tissues, although such effects appear to contribute less to true emergency flare‐ups in some clinical studies (Ehrmann et al. 2003, 2007). Finally, host‐related factors—including systemic conditions and psychosocial variables such as anxiety—may modulate the magnitude of inflammation and nociceptive processing, thereby influencing whether postoperative symptoms escalate to an unscheduled emergency visit (Fouad and Burleson 2003; Law et al. 2015).

Several previous reviews have addressed postoperative pain and/or flare‐ups after root canal treatment (e.g., Tsesis et al. 2008; Pak and White 2011; Vishwanathaiah et al. 2021); however, many studies included in these reviews used heterogeneous or relatively broad outcome definitions, making it difficult to specifically interpret the evidence for clinically significant flare‐ups. Moreover, prior syntheses often focused on selected procedural comparisons (e.g., single‐ vs. multiple‐visit treatment) rather than comprehensively evaluating predictors. Therefore, an updated synthesis using a strict, clinically relevant flare‐up definition and contemporary risk‐of‐bias and certainty‐of‐evidence frameworks was deemed necessary.

Given this background, this systematic review and meta‐analysis aimed to identify and quantify risk factors associated with clinically significant endodontic flare‐ups, defined as postoperative pain and/or swelling within approximately 1 week of root canal treatment requiring an unscheduled emergency visit and additional treatment (Siqueira 2003). Because flare‐ups typically occur within 24–48 h after treatment, but postoperative pain is commonly assessed for up to 7 days, we adopted a conservative postoperative time window of approximately 1 week for study identification (Tsesis et al. 2008; Mendes et al. 2025). The review question formulated in accordance with the PICO framework was as follows: In patients undergoing root canal treatment, does the presence of specific patient‐, tooth‐, preoperative symptom‐, or treatment‐related factors increase the risk of endodontic flare‐ups compared with their absence? We synthesized the available evidence using pooled relative risks and assessed certainty using contemporary risk‐of‐bias tools and the GRADE approach.

## 2 Materials and Methods

### 2.1 Literature Search

Prior to initiation of the study, the protocol was registered in the PROSPERO international prospective registry for systematic reviews (CRD420251025451) and conducted in accordance with the Preferred Reporting Items for Systematic Reviews and Meta‐Analyses (PRISMA) statement and checklist (Tables S1 and S2) (Page et al. 2021).

A comprehensive literature search was conducted in three electronic databases—PubMed, Scopus, and Web of Science—with the final search performed on 30 September 2025. To minimize the risk of missing relevant studies, a manual search was undertaken in two major endodontic journals (Journal of Endodontics and International Endodontic Journal). In addition, the reference lists of key systematic reviews and frequently cited original studies identified through the initial electronic search were screened. Grey literature was searched using ProQuest Dissertations & Theses Global. All searches were performed independently by two endodontists (J.O. and M.M.). Medical Subject Headings (MeSH) and relevant keywords were combined using the Boolean operators “AND” and “OR” to construct the search strategy. Retrieved records were manually screened to identify and remove duplicates. No restrictions were applied regarding language, year of publication, or country of origin. The detailed search strategies are provided in the Supporting Information Methods.

### 2.2 Study Selection

Two reviewers (J.O. and M.M.) independently screened the titles and abstracts of all records identified through the electronic and manual searches. Studies meeting the predefined inclusion criteria were selected for full‐text review. Full texts of potentially eligible articles were obtained and assessed independently by both reviewers. Disagreements at any stage were resolved through discussion; when consensus could not be achieved, a third reviewer (Y.Ka.) served as an adjudicator. The inclusion criteria were as follows:

1. Observational or interventional studies published up to September 2025 that reported the occurrence of flare‐ups after root canal treatment as the primary outcome.
2. Studies defining a flare‐up as “pain and/or swelling requiring an unscheduled emergency visit and additional treatment within approximately 1 week after treatment,” including inter‐appointment events.
3. Studies providing clearly documented data on potential risk factors.

The exclusion criteria were as follows:

1. Reviews, case reports, animal studies, or in vitro studies.
2. Studies not addressing flare‐ups associated with root canal treatment.
3. Studies focusing on paediatric patients or immature teeth.

### 2.3 Quality Assessment and Data Extraction

Two reviewers (J.O. and M.M.) independently extracted data from each included study. The following variables were collected: author (year), country, study design, sample size, definition of flare‐up, risk factors assessed, incidence of flare‐up, statistical methods used, and risk factors identified as statistically significant. In cases of disagreement regarding extracted information, a third reviewer (Y.Ka.) mediated to achieve consensus. Studies with partially reported or unclear data were retained; however, only clearly documented and extractable variables were included in the quantitative synthesis. Among the broad range of potential predictors assessed, only those repeatedly examined and comparably defined across multiple studies were included in the meta‐analysis, resulting in 13 eligible risk factors.

The risk of bias for each included study was assessed using the most recent version of the Cochrane risk‐of‐bias tools appropriate to the study design. For randomized controlled trials (RCTs), the Risk of Bias 2.0 (RoB 2.0) tool was applied, and risk was judged on a three‐level scale (low risk, some concerns, high risk) across five domains: (1) appropriateness of randomization, (2) adherence to the intended intervention, (3) missing outcome data, (4) fairness of outcome measurement, and (5) selective reporting of results. For non‐randomized studies, the revised 2024 version of ROBINS‐I V2 (Risk Of Bias In Non‐Randomized Studies—of Interventions) was used. Each domain was rated as low, moderate, serious, or critical risk of bias, with the overall judgement corresponding to the highest domain‐level risk. The following domains were evaluated: (1) confounding, (2) participant selection, (3) exposure classification, (4) deviations from intended exposure/interventions, (5) missing data, (6) outcome measurement, and (7) selective reporting. To improve transparency and consistency, review‐specific operational criteria were applied across all included non‐randomized studies.

In the confounding domain, particular attention was paid to whether key prognostic factors—especially preoperative spontaneous pain and/or percussion pain, pulp vitality/non‐vital status, periapical radiolucency, retreatment status, and number of treatment visits—were appropriately accounted for. Studies using multivariable analyses and adequately addressing most of these factors were judged at low or moderate risk, whereas studies relying mainly on crude comparisons or without statistical control for important confounders were judged at serious risk. For participant selection, consecutive or clearly defined clinical cohorts were judged more favourably than studies with insufficiently described sampling procedures. Exposure classification was judged according to the clarity and objectivity of record‐ or chart‐based definitions for variables such as retreatment status, tooth location, tooth type, pulp status, and visit number. Bias due to deviations from intended exposures/interventions was generally considered low in these observational studies because the assessed exposures reflected recorded clinical characteristics or treatment conditions rather than investigator‐assigned interventions. For missing data, studies with explicit evidence of complete or near‐complete ascertainment were judged at lower risk, whereas studies with no clear description of missingness or follow‐up completeness were judged at moderate risk. Outcome measurement was judged according to the clinical specificity and consistency of the flare‐up definition, with lower risk assigned when flare‐up was defined as severe pain and/or swelling requiring an unscheduled visit and additional treatment within a clearly defined postoperative period. Selection of the reported result was judged according to whether sufficient data were available for the planned syntheses and whether selective emphasis on significant findings was apparent. Overall ROBINS‐I judgements were based on the highest level of risk observed across domains. Detailed review‐specific decision rules are provided in Table S3.

All risk‐of‐bias assessments were conducted independently by J.O. and M.M., with final judgements reached through discussion. When discrepancies persisted, Y.Ka. acted as mediator, and consensus was achieved. Graphical summaries of the risk‐of‐bias assessments were generated using ROBVIS, a web‐based application built on RStudio (McGuinness and Higgins 2021).

### 2.4 Outcome Variables and Statistical Analysis

In this study, relative risks (RRs) were used to evaluate the association between each risk factor and the incidence of flare‐ups. When necessary, RRs were recalculated directly from reported 2 × 2 event data (including studies that originally presented odds ratios) to ensure a consistent effect measure across studies. An RR of 1 indicates no difference in flare‐up risk between groups, an RR of > 1 indicates increased risk in the exposed group, and an RR of < 1 indicates decreased risk. For pooled analyses, we applied a random‐effects model using the DerSimonian‐Laird method to account for between‐study variability, under the assumption that clinical heterogeneity was likely given differences in study settings and populations. Effect estimates were pooled on the log RR scale using the inverse‐variance method and are presented as forest plots (Lewis 2001). Statistical heterogeneity was evaluated using Cochran's Q test and quantified with the *I* ^2^ statistic. *I* ^2^ values of approximately 25%, 50%, and 75% were considered low, moderate, and high heterogeneity, respectively (Higgins et al. 2003). When substantial heterogeneity (*I* ^2^ ≥ 50%) was observed, pooled estimates were interpreted cautiously, and potential sources were explored by comparing study characteristics and conducting prespecified sensitivity analyses. For outcomes with ≥ 10 studies, we assessed small‐study effects by visual inspection of funnel plots and tested using Egger's regression (Sterne and Egger 2001), implemented in EZR software (Kanda 2013). We also conducted exploratory univariable random‐effects meta‐regression (when sufficient studies were available) to evaluate whether study‐level prevalence of prespecified characteristics explained between‐study variability, including the proportions of participants aged ≥ 50 years, female participants, mandibular teeth, teeth with apical radiolucency, re‐treatment cases, and multiple‐visit treatments. Meta‐regression was performed on the log RR scale using inverse‐variance weighting. Statistical significance was accepted at a *p* value of < 0.05. All statistical analyses were performed using RevMan version 5.3 software.

### 2.5 Quality Evidence Assessment

To assess the certainty of the evidence, we used the GRADE evaluation system (Brennan and Johnston 2023). The GRADE tool includes five domains—risk of bias, inconsistency, imprecision, indirectness, and publication bias—that can be downgraded when there is concern that the quality of evidence may be reduced (Guyatt et al. 2011). The GRADE evidence profile and summary of findings tables were created using GRADEpro software.

## 3 Results

### 3.1 Search Results

**FIGURE 1:**
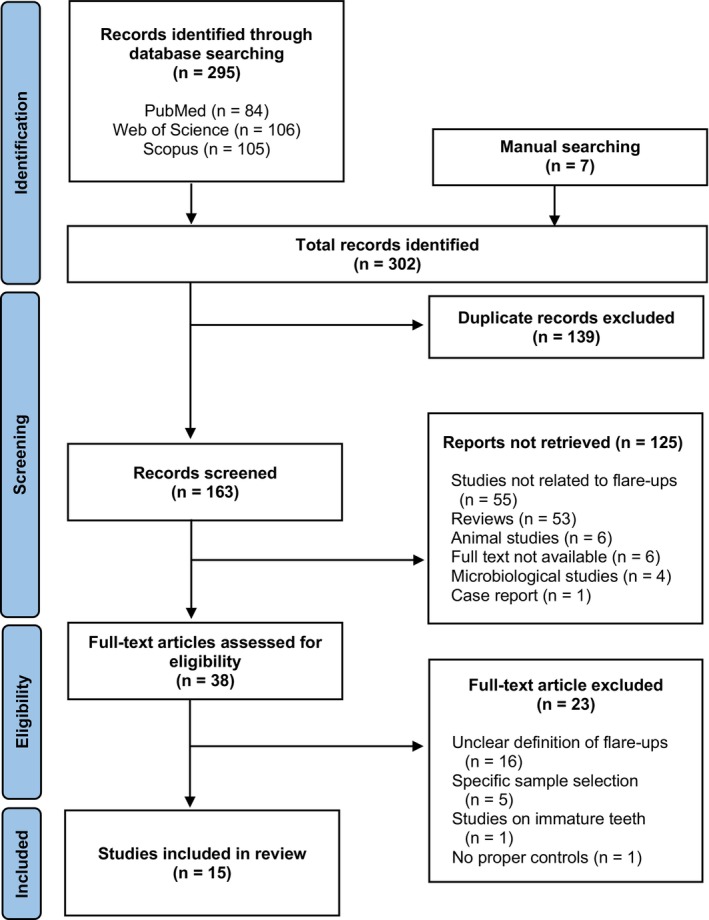
Flow diagram of study selection for the systematic review and meta‐analysis.

In total, 302 articles were identified through electronic database and manual searches. No additional eligible studies were identified through the grey literature search. After removing duplicates, 163 abstracts were screened, and 38 papers were selected for full‐text review based on their titles and abstracts. Ultimately, 15 articles met the inclusion criteria and were included in the systematic review and meta‐analysis. Most included studies assessed flare‐ups within 7 days, whereas one study (Nosrat et al. 2023) used a 14‐day window; this study was retained to maintain comparability across the included reports. Of the excluded articles, 16 had ambiguous or unclear definitions of flare‐ups, 5 used highly specific sample selection criteria that limited generalizability (e.g., restriction to narrowly defined subgroups such as specific tooth types, specific pulpal/periapical conditions, retreatment‐only populations, or highly selected treatment cohorts), 1 addressed root canal treatment of immature teeth, and 1 did not include an appropriate control group (Figure 1).

### 3.2 Study Characteristics

The general characteristics of the included studies are summarized in Table S4. The study designs included 1 RCT, 4 prospective observational studies, and 10 retrospective observational studies. Because a strict, clinically oriented definition of flare‐up was applied, most included studies were retrospective and based on existing clinical records. Sample sizes ranged from 120 to 6580 cases, with a total of 24 320 cases included in the analysis.

### 3.3 Incidence of Flare‐Ups

Flare‐ups occurred in 688 cases (2.83%) overall. Incidence rates in individual studies ranged from 0.39% to 9.40%, depending on the reported risk factors and clinical settings.

### 3.4 Meta‐Analysis

Thirteen risk factors were meta‐analysed where comparable data were available across the included studies: age, sex, tooth position, pulp condition, preoperative spontaneous pain, periapical lesions, number of treatment visits, initial treatment versus re‐treatment, number of root canals, instrumentation type (NiTi rotary versus stainless steel hand files), preoperative percussion pain, tooth type, and preoperative analgesic use.

Age was analysed in two categories (≤ 49 years and ≥ 50 years), because these groupings were frequently used in individual studies (e.g., Imura and Zuolo 1995; Walton and Fouad 1992). The pooled RR for flare‐up incidence in patients aged ≥ 50 years was 1.30 (95% confidence interval [CI]: 0.84–2.00), and the certainty of evidence for the age effect was rated as very low, with substantial heterogeneity (*I* ^2^ = 71%; Q‐test *p* = 0.0002) (Figure S1A). Egger's test did not indicate funnel plot asymmetry (Figure S1B). Exploratory univariable meta‐regression suggested that higher proportions of periapical lesions were associated with a smaller apparent age effect (*β* = −4.89 on the log RR scale; *p* = 0.032) (Table S5).

**FIGURE 2:**
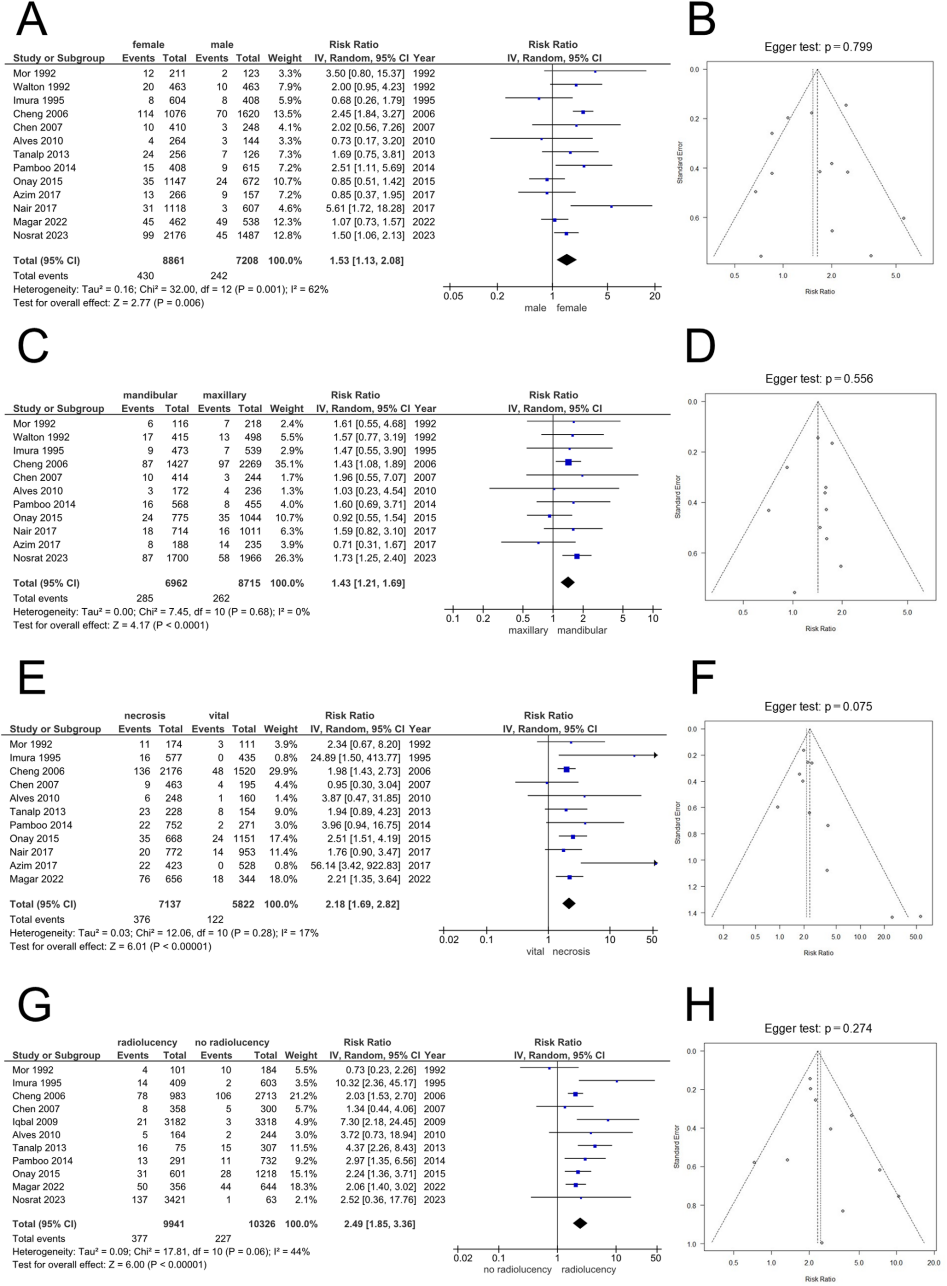
Forest and funnel plots showing the association of sex, tooth position (mandible vs. maxilla), pulp status (non‐vital vs. vital), and periapical radiolucency with flare‐up incidence. Forest plots comparing flare‐up incidence by sex (A), tooth position (C), pulp status (E), and periapical radiolucency (G); and funnel plots assessing publication bias for sex (B), tooth position (D), pulp status (F), and periapical radiolucency (H).

Women had a significantly higher incidence of flare‐ups than men, with a pooled RR of 1.53 (95% CI: 1.13–2.08) (Figure 2A), with moderate heterogeneity (*I* ^2^ = 62%; Q‐test *p* = 0.001) and no evidence of funnel plot asymmetry (Figure 2B). Sensitivity analyses (leave‐one‐out and exclusion of high‐risk‐of‐bias studies) did not change the direction of the effect. Exploratory univariable meta‐regression did not identify study‐level covariates associated with the sex effect (all *p* ≥ 0.22) (Table S5).

With respect to tooth location, flare‐ups were more frequent in mandibular than in maxillary teeth (pooled RR = 1.43; 95% CI: 1.21–1.69) (Figure 2C). Between‐study heterogeneity was negligible (*I* ^2^ = 0%), and Egger's regression test did not suggest funnel plot asymmetry (Figure 2D).

Non‐vital teeth were associated with a higher risk of flare‐ups than were vital teeth (pooled RR = 2.18; 95% CI: 1.69–2.82) (Figure 2E). Heterogeneity was low (*I* ^2^ = 17%), suggesting generally consistent findings across studies. Egger's regression test did not show statistically significant funnel plot asymmetry, although the result was borderline (*p* = 0.075), and small‐study effects cannot be entirely excluded (Figure 2F).

Apical radiolucency was associated with an increased risk of flare‐ups (pooled RR = 2.49; 95% CI: 1.85–3.36) (Figure 2G). Moderate heterogeneity was observed (*I* ^2^ = 44%, Q‐test *p* = 0.06), and no evidence of funnel plot asymmetry (Figure 2H).

**FIGURE 3:**
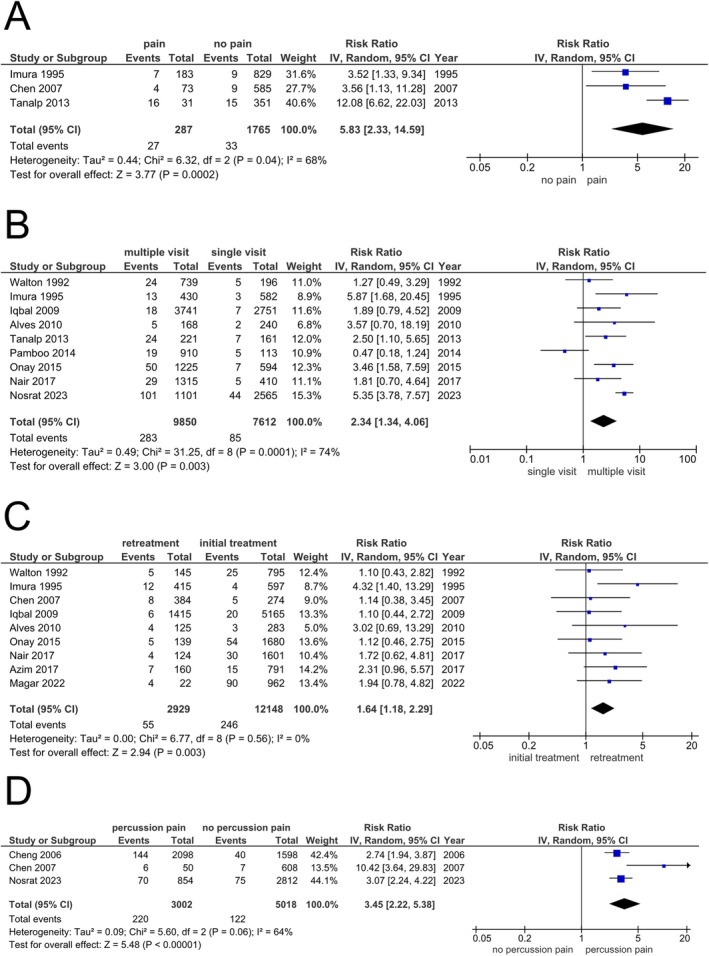
Forest plots showing the association of preoperative and procedural factors with flare‐up incidence. Forest plots comparing flare‐up incidence by preoperative spontaneous pain (A), number of treatments (multiple vs. single visit) (B), treatment type (re‐treatment vs. initial treatment) (C), and preoperative percussion pain (D).

The presence of preoperative spontaneous pain was strongly associated with flare‐ups (pooled RR = 5.83; 95% CI: 2.33–14.59) (Figure 3A). Substantial heterogeneity was observed (*I* ^2^ = 68%; Q‐test *p* = 0.04), and the wide CI indicated imprecision in the pooled estimate, despite a consistent direction of association. Sensitivity analyses did not change the direction of the association, although the magnitude of the pooled RR varied.

Multiple‐visit treatments were associated with a higher risk of flare‐ups than single‐visit treatments, with a pooled RR of 2.34 (95% CI: 1.34–4.06) (Figure 3B). Substantial heterogeneity was present (*I* ^2^ = 74%; Q‐test *p* = 0.0001), reflecting considerable between‐study variability in effect size. Leave‐one‐out analyses did not alter the direction of the association, although the pooled estimate varied.

Re‐treatment cases demonstrated a higher frequency of flare‐ups than did initial treatment, with a pooled RR of 1.64 (95% CI: 1.18–2.29) (Figure 3C). Heterogeneity was negligible (*I* ^2^ = 0%).

Preoperative percussion pain was significantly associated with flare‐ups, with a pooled RR of 3.45 (95% CI: 2.22–5.38) (Figure 3D). Moderate‐to‐substantial heterogeneity was observed (*I* ^2^ = 64%; Q‐test *p* = 0.06). Leave‐one‐out analyses did not change the direction of the association, although the magnitude of the pooled estimate varied.

By contrast, no association was observed with the number of root canals (pooled RR = 0.99; 95% CI: 0.74–1.33) (Figure S1C). No significant difference was found between NiTi rotary and stainless steel hand instrumentation, with a pooled RR of 1.85 (95% CI: 0.82–4.16) (Figure S1D). Although the point estimate suggested a higher flare‐up risk in the stainless steel group, the CI was wide and crossed unity, indicating substantial uncertainty. Preoperative analgesic use was associated with a pooled RR of 2.50 (95% CI: 0.68–9.18); however, the estimate was highly imprecise, and between‐study heterogeneity was substantial (*I* ^2^ = 85%; Q‐test *p* = 0.001) (Figure S1E).

Tooth type was evaluated in 12 studies. No significant differences were observed when anterior teeth were compared with other teeth (pooled RR = 1.06, 95% CI: 0.85–1.31) (Figure S2A,B), or when premolars were compared with other teeth (pooled RR = 0.85, 95% CI: 0.67–1.08) (Figure S2C,D). For molars, the pooled estimate suggested a higher risk (RR = 1.21; I^2^ = 0%) (Figure S2E,F). Under the conventional DerSimonian–Laird model, the 95% CI marginally excluded 1 (1.001–1.457); however, this borderline statistical significance was not maintained with the Hartung–Knapp adjustment (95% CI: 0.991–1.472). Given the limited number of contributing studies, the Hartung–Knapp method was considered more conservative (Röver et al. 2015). Therefore, the evidence for increased risk in molars was not robust.

Overall, these findings indicate that female sex, mandibular tooth location, non‐vital pulp status, preoperative spontaneous pain, the presence of periapical lesions, multiple‐visit treatment, re‐treatment, and preoperative percussion pain are significantly associated with flare‐ups (Table 1). However, given between‐study heterogeneity and imprecision in several estimates, strong causal influences cannot be made for many of these factors.

**TABLE 1:** Pooled relative risks for clinically significant endodontic flare‐ups.

Risk factor	Pooled RR (95% CI)	Association	Figure
Preoperative spontaneous pain	5.83 (2.33–14.59)	↑	Figure 3A
Preoperative percussion pain	3.45 (2.22–5.38)	↑	Figure 3D
Apical radiolucency	2.49 (1.85–3.36)	↑	Figure 2G
Multiple‐visit treatment	2.34 (1.34–4.06)	↑	Figure 3B
Non‐vital pulp	2.18 (1.69–2.82)	↑	Figure 2E
Re‐treatment	1.64 (1.18–2.29)	↑	Figure 3C
Female sex	1.53 (1.13–2.08)	↑	Figure 2A
Mandibular tooth location	1.43 (1.21–1.69)	↑	Figure 2C
Tooth type: molar vs. others	1.21 (1.00–1.46)*	↑^a^	Figure S2E

*Note:* Association: ↑ indicates increased risk.

Abbreviations: CI, confidence interval; RR, relative risk.

* Under the conventional DerSimonian–Laird random‐effects model, the 95% CI marginally excluded 1.

^a^ The borderline association was sensitive to the variance estimator and was not maintained under the Hartung–Knapp adjustment; therefore, it was interpreted as not robust.

### 3.5 Risk of Bias and Quality Evidence Assessment

**FIGURE 4:**
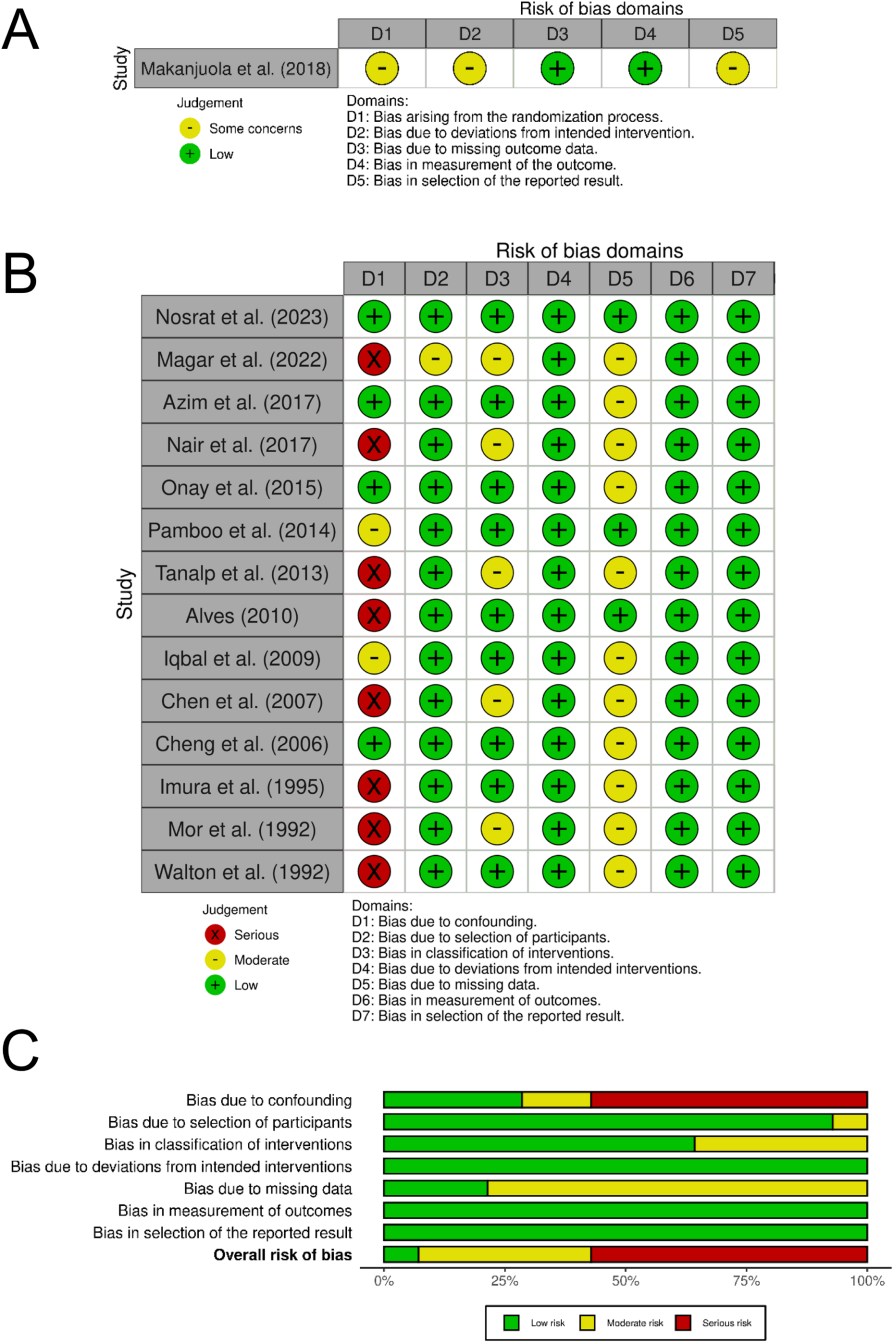
Risk of bias assessment for included studies. (A) Risk of bias assessment for the randomized controlled trial (RCT) using RoB2.0. (B) Risk of bias assessment for non‐RCTs using ROBINS‐I‐V2. (C) Summary of risk of bias across the 14 non‐RCTs.

Risk of bias was assessed separately for RCTs and non‐RCTs using the appropriate tools. The single RCT included in the analysis (Makanjuola et al. 2018) was judged as having “some concerns” according to the RoB 2.0 tool (Figure 4A). Among the 14 non‐RCTs, ROBINS‐I‐V2 assessments classified only one study as low risk of bias (Nosrat et al. 2023), five at moderate risk (Azim et al. 2017; Cheng et al. 2006; Iqbal et al. 2009; Onay et al. 2015; Pamboo et al. 2014), and eight at serious risk (Alves 2010; Chen et al. 2007; Imura and Zuolo 1995; Magar et al. 2022; Mor et al. 1992; Nair et al. 2017; Tanalp et al. 2013; Walton and Fouad 1992) (Figure 4B,C). Using GRADE, the certainty of evidence for several risk factors was low or very low (Table 2 and Table S6), largely due to risk of bias and study limitations.

**TABLE 2:** GRADE assessment of the certainty of evidence for factors associated with flare‐up incidence.

Certainty assessment							Certainty	Importance
No. of studies	Study design	Risk of bias	Inconsistency	Indirectness	Imprecision	Other considerations		
Relationship between sex (female) and flare‐ups								
13	Non‐randomized studies	Serious^a^	Serious^b^	Not serious	Not serious	None	⨁⨁◯◯ Low^a^, ^b^	Not important
Relationship between the jaw area (mandibular) and flare‐ups								
11	Non‐randomized studies	Serious^a^	Not serious	Not serious	Not serious	None	⨁⨁⨁◯ Moderate^a^	Important
Relationship between dental pulp condition (non‐vital teeth) and flare‐ups								
11	Non‐randomized studies	Serious^a^	Not serious	Not serious	Not serious	None	⨁⨁⨁◯ Moderate^a^	Important
Relationship between preoperative spontaneous pain and flare‐ups								
3	Non‐randomized studies	Serious^a^	Serious^c^	Not serious	Serious^d^	None	⨁◯◯◯ Very low^a^, ^c^, ^d^	Not important
Relationship between periapical lesions and flare‐ups								
11	Non‐randomized studies	Serious^a^	Serious^e^	Not serious	Not serious	None	⨁⨁◯◯ Low^a^, ^e^	Not important
Relationship between the number of treatments (multiple times) and flare‐ups								
9	Non‐randomized studies	Serious^a^	Serious^f^	Not serious	Not serious	None	⨁⨁◯◯ Low^a^, ^f^	Not important
Relationship between re‐treatment and flare‐ups								
9	Non‐randomized studies	Serious^a^	Not serious	Not serious	Not serious	None	⨁⨁⨁◯ Moderate^a^	Important
Relationship between preoperative percussion pain and flare‐ups								
3	Non‐randomized studies	Serious^a^	Serious^g^	Not serious	Not serious	None	⨁⨁◯◯ Low^a^, ^g^	Not important

^a^ In the ROBINS‐I‐v2 evaluation, many studies are rated as “moderate” or “serious”.

^b^ *I* ^2^ = 62.5%, Q = 32.0 (df = 12, *p* = 0.001).

^c^ *I* ^2^ = 68.4%, Q = 6.32 (df = 2, *p* = 0.042).

^d^ RR is very high at 5.83, but the 95% CI was very wide at 2.33–14.59. The number of studies is small, at only three, and there are concerns about the reliability of the estimates.

^e^ *I* ^2^ = 43.9%, Q = 17.81 (df = 10, *p* = 0.058).

^f^ *I* ^2^ = 74.4%, Q = 31.25 (df = 8, *p* = 0.0001).

^g^ *I* ^2^ = 64.3%, Q = 5.60 (df = 2, *p* = 0.061).

## 4 Discussion

This systematic review and meta‐analysis identified and quantitatively assessed risk factors associated with clinically significant endodontic flare‐ups using our predefined definition. Fifteen studies (24 320 cases) were included, and the overall incidence of flare‐ups was 2.83%.

Several factors were significantly associated with an increased risk of flare‐up after root canal treatment. In particular, the strong associations observed for non‐vital pulp status, apical radiolucency, and preoperative pain indicators (spontaneous pain and percussion pain) highlight the central role of pre‐existing infection and inflammation in flare‐up pathogenesis. Necrotic pulps and radiographically evident apical periodontitis typically involve complex intra‐ and extra‐radicular biofilms with high bacterial loads and diverse microbial communities with substantial inflammatory potential (Siqueira and Rôças 2022). In such cases, chemomechanical preparation may dislodge and extrude bacterial aggregates, toxins, or necrotic debris into the periapical tissues, abruptly intensifying the inflammatory response and increasing tissue pressure (Seltzer and Naidorf 2004b). This interpretation is consistent with previous clinical evidence indicating that higher preoperative pain and presence of apical periodontitis are associated with increased postoperative pain and flare‐ups, plausibly reflecting an advanced inflammatory state and peripheral nociceptor sensitization (Aoun et al. 2019; Genet et al. 1986; Silva et al. 2022). Preoperative spontaneous pain and percussion pain may also be regarded as clinical correlates of hyperalgesia/allodynia in sensitized periapical tissues, where even routine endodontic procedures can trigger disproportionate nociceptive responses and acute symptom escalation (Erdogan et al. 2019). Clinically, careful assessment and documentation of preoperative pain characteristics may therefore represent one of the most practical approaches to identifying patients at higher risk of flare‐ups.

Several associations likely reflect underlying treatment complexity and case selection in routine practice rather than direct causal effects of procedural choices alone. The increased risk in re‐treatment cases is biologically plausible because these teeth often present with persistent infection, altered anatomy, procedural complications, and compromised periapical tissues, all of which increase treatment difficulty and may heighten postoperative symptom exacerbation (Torabinejad et al. 1988).

The association between multiple‐visit treatment and flare‐ups should be interpreted cautiously. Although multi‐visit strategies are often selected for complex cases, inter‐appointment procedures and materials could aggravate already inflamed periapical tissues in susceptible patients. However, discrepancies from RCT or prospective evidence may also reflect outcome timing: postoperative pain is often assessed only after obturation, and inter‐appointment symptoms are frequently not counted, whereas observational records capture unscheduled visits during multi‐visit care and may classify them as flare‐ups (Izadpanah et al. 2021; Schwendicke and Göstemeyer 2017; Vishwanathaiah et al. 2021). Thus, confounding by indication and differential ascertainment likely contribute to the observed association.

Regarding tooth type, anterior and premolar teeth showed no clear association with flare‐up risk. For molars, the pooled estimate suggested a small increase in risk (RR = 1.21) under the DerSimonian–Laird random‐effects model, but this borderline association was not maintained using the Hartung–Knapp adjustment. This sensitivity to the statistical approach indicates that the evidence for a molar‐specific increase is not robust (Nair et al. 2017; Onay et al. 2015). Any apparent molar effect is also likely confounded by disease severity and case complexity, because molars more frequently present with complex anatomy, necrotic pulps, apical periodontitis, and re‐treatment, which are themselves established predictors of flare‐ups (Bassam et al. 2021). Consistent with this, studies on postoperative endodontic pain have reported mixed findings for tooth type, suggesting that any independent effect of tooth category is modest at best (Aoun et al. 2019; Hegde et al. 2025).

The higher flare‐up risk observed in women likely reflects both biological and psychosocial influences. Sex‐related differences in hormonal modulation of inflammation and nociceptive processing may contribute biologically (Caputi et al. 2022; Fillingim et al. 2009). In addition, greater pain sensitivity and symptom reporting/healthcare‐seeking behaviour among women could increase the likelihood of unscheduled visits, potentially inflating the measured incidence of clinically significant flare‐ups (Racine et al. 2012). Similarly, the consistent association for mandibular tooth position may partly reflect anatomical and host‐environment differences; mandibular bone is generally denser, which may impede drainage and increase inflammatory pressure (Devlin et al. 1998). However, apparent “anatomical” effects may also be influenced by unmeasured clinical factors and case‐mix differences across cohorts.

By contrast, age (≥ 50 years), number of root canals, type of file used (NiTi rotary files versus stainless steel hand files), and preoperative analgesic use were not significantly associated with the incidence of flare‐ups. For age, the limited number of contributing studies and the arbitrary dichotomization at 50 years may have obscured more nuanced age‐related patterns, such as potential differences between younger and older patients in pain reporting or immune response.

Although a higher canal count could plausibly increase opportunities for apical debris extrusion during negotiation, instrumentation, and irrigation (Tanalp and Güngör 2014), our meta‐analysis did not show a clear independent association with flare‐up risk. This may reflect confounding by tooth type and disease severity (e.g., pulp/periapical status, apical radiolucency, and retreatment), which could attenuate any canal‐number effect after adjustment (Gulabivala and Ng 2023; Ng et al. 2011). In addition, canal counts are a crude and potentially misclassified proxy, and the low event rate may limit power to detect small independent effects (Ioannidis 2005). Similarly, the lack of a clear difference between NiTi rotary and hand instrumentation suggests that, when contemporary protocols are used, the choice of file system per se may be less important than overall treatment quality and apical control of preparation and irrigation. However, these null findings should be interpreted cautiously, considering wide CIs and the low or very low certainty of evidence.

Substantial heterogeneity across several analyses likely reflects both methodological and clinical differences between studies. Variability in access to care, help‐seeking behaviour, and record completeness can affect ascertainment of unscheduled emergency visits, while cohorts differed in baseline disease severity and case‐mix (e.g., necrosis, apical radiolucency, retreatment, and preoperative pain), which may modify observed effects. In observational data, confounding by indication is also likely (e.g., multiple‐visit treatment selected for more complex cases). Finally, the low event rate (~2%–3%) and few contributing studies for some comparisons can yield unstable estimates and wide confidence intervals.

To explore potential sources of heterogeneity, we conducted exploratory univariable random‐effects meta‐regression analyses using prespecified study‐level covariates (Table S5). These analyses were designed to assess whether differences in case mix might partly explain between‐study variability. For age, a higher prevalence of apical radiolucency was associated with a smaller apparent age effect, suggesting that underlying disease severity may overshadow age‐related differences. By contrast, for other predictors, examined covariates did not account for the observed effects, possibly due to unmeasured protocol/operator factors and variation in outcome ascertainment. These findings should be interpreted cautiously as hypothesis‐generating, given limited power and ecological bias.

A major strength of this study is its rigorous methodology, including a comprehensive search and a strict, clinically oriented flare‐up definition focusing on severe events requiring unscheduled care. In contrast, Tsesis et al. reported a higher incidence (8.4%) in a review largely based on RCTs and prospective series, which may reflect broader outcome definitions and different ascertainment methods (Tsesis et al. 2008). The large cumulative sample size (*n* = 24 320) enabled pooling for multiple major risk factors, and inclusion of studies across diverse geographic regions may enhance generalizability. Many of the identified risk factors have also been highlighted in Monte Carlo simulation–based models, aligning with our results (Aksoy et al. 2021). This convergence between empirical data and simulation approaches supports their potential importance as predictors of flare‐ups.

At the same time, the strictness of the adopted definition resulted in the inclusion of predominantly retrospective studies, a relatively limited number of eligible studies, and the potential influence of confounding and selection bias, which represent important limitations. Treatment protocols, operator experience, and documentation practices varied across settings. For several comparisons, the number of contributing studies was small, resulting in wide confidence intervals and low/very low certainty of evidence. Additional potentially relevant factors (e.g., irrigant regimens, microbiological profiles, and systemic conditions) could not be evaluated consistently due to limited reporting.

Nosrat et al. (2023) was the only included study rated low overall risk of bias and provided the most methodologically robust evidence currently available; its findings were broadly consistent with our pooled estimates, particularly for preoperative pain indicators (Nosrat et al. 2023). Despite large effect estimates for spontaneous and percussion pain, GRADE certainty remained low to very low because certainty reflects overall evidence reliability rather than statistical significance and was downgraded for risk of bias, heterogeneity, and imprecision. Future large‐scale prospective studies with standardized definitions and rigorous confounding control are warranted to strengthen the certainty of these associations.

## 5 Conclusion

In this systematic review and meta‐analysis of 15 studies (24 320 cases), clinically significant endodontic flare‐ups occurred in 2.83% of treatments. The most consistent and clinically important predictors were markers of pre‐existing infection and inflammation—non‐vital pulp, apical radiolucency, and particularly preoperative pain. Spontaneous pain (RR 5.83) and percussion pain (RR 3.45) conferred the highest risks, highlighting preoperative pain assessment as a practical means to identify high‐risk cases and to guide anticipatory counselling and proactive pain management. Higher risk was also observed for mandibular teeth, retreatment, and multiple‐visit treatment, although these associations may reflect case complexity and differential outcome ascertainment. Given the overall low certainty of evidence, well‐designed prospective studies using standardized flare‐up definitions are warranted.

## Author Contributions

Conceptualization: J.O. and H.M. Data Curation: J.O., M.M., and Y.Ka. Formal analysis: J.O., M.M., Y.Ka., S.A., N.T., T.S., and H.M. Funding acquisition: J.O., M.M., S.A., H.M., and M.H. Methodology: J.O., Y.Ki., and M.H. Supervision: J.O., Y.Ki., and M.H. Validation: J.O., M.M., and Y.Ka. Visualization: J.O., Y.Ka., and H.M. Writing – original draft preparation: J.O., M.M., and Y.Ka. Writing – review and editing: J.O., Y.Ki., and M.H. All authors gave final approval and agreed to be accountable for all aspects of the work.

## Funding

This work was supported by Grants‐in‐Aid for Scientific Research from the Japan Society for the Promotion of Science: 22K09980 to J. Ohshima, 24K02620 and 24K22184 to M. Hayashi, 22KJ2187 and 26K20197 to M. Morita, 24K19878 and 26K20175 to S. Abe, 26K20198 to T. Shimaoka, and 25K13003 to H. Maezono.

## Ethics Statement

The authors have nothing to report.

## Consent

The authors have nothing to report.

## Conflicts of Interest

The authors declare no conflicts of interest.

## Supporting information


**Figure S1:** Forest and funnel plots showing the association of other clinical factors with flare‐up occurrence. Forest plots comparing flare‐up occurrence by age (≥ 50 years vs. ≤ 49 years) (A), number of root canals (C), type of file used (NiTi rotary vs. stainless steel hand files) (D), and preoperative analgesic use (E); and funnel plot assessing publication bias for age (B).
**Figure S2:** Forest and funnel plots showing the association of tooth type with flare‐up occurrence. Forest plots comparing flare‐up occurrence between anterior teeth and other teeth (A), premolar teeth and other teeth (C), and molar teeth and other teeth (E); and funnel plots assessing publication bias for anterior teeth (B), premolar teeth (D), and molar teeth (F).
**Table S1:** PRISMA2020 Checklist.
**Table S2:** PRISMA2020 for Abstracts Checklist.
**Table S3:** Review‐specific operational criteria for ROBINS‐I‐V2 judgements in non‐randomized studies.
**Table S4:** Study characteristics of the included articles.
**Table S5:** Univariable meta‐regression analyses exploring study‐level effect modifiers of pooled associations with endodontic flare‐ups.
**Table S6:** GRADE assessment of the certainty of evidence for additional risk factors.

## Data Availability

The data that support the findings of this study are available from the corresponding author upon reasonable request.
