# Supplementary material for: Factors Associated With Endodontic Flare‐Ups: A Systematic Review and Meta‐Analysis
Source: Int Endod J. 2026 Apr 23;59(9):1778–91. doi: 10.1111/iej.70164 (PMC13462396; doi:10.1111/iej.70164)
Supplement: Supplementary file 1 [file IEJ-59-1778-s001.docx]

**Factors associated with endodontic flare-ups: A systematic review and meta-analysis**

Running title: **ENDODONTIC FLARE-UP FACTORS**

**SUPPLEMENTARY METHODS**

**Search strategy in electronic databases**

A comprehensive literature search was conducted in PubMed, Scopus, and Web of Science to identify studies related to potential risk factors associated with flare-ups or interappointment emergencies in endodontics. The searches were conducted on 30 September 2025. No filters for publication year or study design were applied. The full search strings used in each database were as follows:

**PubMed**:

((variable* OR parameter* OR predictor* OR factor OR factors OR risk) AND (flareup* OR flare-up* OR "flare up" OR "flare ups" OR "interappointment emergency" OR "inter appointment emergency") AND ("root canal" OR "root canal therapy" OR "root canal treatment" OR endodontic*))

**Scopus**:

TITLE-ABS-KEY(variable* OR parameter* OR predictor* OR factor OR factors OR risk)　AND TITLE-ABS-KEY(flareup* OR flare-up* OR "flare up" OR "flare ups" OR "inter appointment emergenc*" OR "interappointment emergenc*") AND TITLE-ABS-KEY("root canal" OR endodontic*)

**Web of Science**:

TS=(variable* OR parameter* OR predictor* OR factor OR factors OR risk) AND TS=(flareup* OR flare-up* OR "flare up" OR "flare ups" OR "interappointment emergenc*" OR "inter appointment emergenc*") AND TS=("root canal" OR "root canal therapy" OR "root canal treatment" OR endodontic*)

**Figure S1**

**Figure S1.**
Forest and funnel plots showing the association of other clinical factors with flare-up occurrence. Forest plots comparing flare-up occurrence by age (≥50 years vs. ≤49 years) (A), number of root canals (C), type of file used (NiTi rotary vs. stainless steel hand files) (D), and preoperative analgesic use (E); and funnel plot assessing publication bias for age (B).

**Figure S2.**

**Figure S2.**

Forest and funnel plots showing the association of tooth type with flare-up occurrence. Forest plots comparing flare-up occurrence between anterior teeth and other teeth (A), premolar teeth and other teeth (C), and molar teeth and other teeth (E); and funnel plots assessing publication bias for anterior teeth (B), premolar teeth (D), and molar teeth (F).

**Table S1.** PRISMA2020 Checklist.

| **Section and Topic** | **Item #** | **Checklist item** | **Location where item is reported** |
| --- | --- | --- | --- |
| **TITLE** | | |  |
| Title | 1 | Identify the report as a systematic review. | Title |
| **ABSTRACT** | | |  |
| Abstract | 2 | See the PRISMA 2020 for Abstracts checklist. | Abstract |
| **INTRODUCTION** | | |  |
| Rationale | 3 | Describe the rationale for the review in the context of existing knowledge. | Introduction - Paragraphs 1-4 |
| Objectives | 4 | Provide an explicit statement of the objective(s) or question(s) the review addresses. | Introduction - Final paragraph |
| **METHODS** | | |  |
| Eligibility criteria | 5 | Specify the inclusion and exclusion criteria for the review and how studies were grouped for the syntheses. | Methods - Study Selection |
| Information sources | 6 | Specify all databases, registers, websites, organisations, reference lists and other sources searched or consulted to identify studies. Specify the date when each source was last searched or consulted. | Methods - Literature Search |
| Search strategy | 7 | Present the full search strategies for all databases, registers and websites, including any filters and limits used. | Appendix - Search Strategy |
| Selection process | 8 | Specify the methods used to decide whether a study met the inclusion criteria of the review, including how many reviewers screened each record and each report retrieved, whether they worked independently, and if applicable, details of automation tools used in the process. | Methods - Study Selection |
| Data collection process | 9 | Specify the methods used to collect data from reports, including how many reviewers collected data from each report, whether they worked independently, any processes for obtaining or confirming data from study investigators, and if applicable, details of automation tools used in the process. | Methods - Quality Assessment and Data Extraction |
| Data items | 10a | List and define all outcomes for which data were sought. Specify whether all results that were compatible with each outcome domain in each study were sought (e.g. for all measures, time points, analyses), and if not, the methods used to decide which results to collect. | Methods - Outcome Variables and Statistical Analysis |
|  | 10b | List and define all other variables for which data were sought (e.g. participant and intervention characteristics, funding sources). Describe any assumptions made about any missing or unclear information. | Methods - Quality Assessment and Data Extraction |
| Study risk of bias assessment | 11 | Specify the methods used to assess risk of bias in the included studies, including details of the tool(s) used, how many reviewers assessed each study and whether they worked independently, and if applicable, details of automation tools used in the process. | Methods - Quality Assessment and Data Extraction |
| Effect measures | 12 | Specify for each outcome the effect measure(s) (e.g. risk ratio, mean difference) used in the synthesis or presentation of results. | Methods - Outcome Variables and Statistical Analysis |
| Synthesis methods | 13a | Describe the processes used to decide which studies were eligible for each synthesis (e.g. tabulating the study intervention characteristics and comparing against the planned groups for each synthesis (item #5)). | Methods - Study Selection |
|  | 13b | Describe any methods required to prepare the data for presentation or synthesis, such as handling of missing summary statistics, or data conversions. | Methods - Outcome Variables and Statistical Analysis |
|  | 13c | Describe any methods used to tabulate or visually display results of individual studies and syntheses. | Methods - Outcome Variables and Statistical Analysis |
|  | 13d | Describe any methods used to synthesize results and provide a rationale for the choice(s). If meta-analysis was performed, describe the model(s), method(s) to identify the presence and extent of statistical heterogeneity, and software package(s) used. | Methods - Outcome Variables and Statistical Analysis |
|  | 13e | Describe any methods used to explore possible causes of heterogeneity among study results (e.g. subgroup analysis, meta-regression). | Methods - Outcome Variables and Statistical Analysis |
|  | 13f | Describe any sensitivity analyses conducted to assess robustness of the synthesized results. | Methods - Outcome Variables and Statistical Analysis |
| Reporting bias assessment | 14 | Describe any methods used to assess risk of bias due to missing results in a synthesis (arising from reporting biases). | Methods - Outcome Variables and Statistical Analysis |
| Certainty assessment | 15 | Describe any methods used to assess certainty (or confidence) in the body of evidence for an outcome. | Methods - Quality Evidence Assessment |
| **RESULTS** | | |  |
| Study selection | 16a | Describe the results of the search and selection process, from the number of records identified in the search to the number of studies included in the review, ideally using a flow diagram. | Results - Search Results, Fig. 1 |
|  | 16b | Cite studies that might appear to meet the inclusion criteria, but which were excluded, and explain why they were excluded. | Results - Search Results, Fig. 1 |
| Study characteristics | 17 | Cite each included study and present its characteristics. | Results - Study Characteristics; Table S3 |
| Risk of bias in studies | 18 | Present assessments of risk of bias for each included study. | Results - Risk of Bias and Quality Evidence Assessment,  Fig. 4 |
| Results of individual studies | 19 | For all outcomes, present, for each study: (a) summary statistics for each group (where appropriate) and (b) an effect estimate and its precision (e.g. confidence/credible interval), ideally using structured tables or plots. | Results - Meta-analysis (Figures and Tables) |
| Results of syntheses | 20a | For each synthesis, briefly summarise the characteristics and risk of bias among contributing studies. | Results - Meta-analysis and Risk of Bias |
|  | 20b | Present results of all statistical syntheses conducted. If meta-analysis was done, present for each the summary estimate and its precision (e.g. confidence/credible interval) and measures of statistical heterogeneity. If comparing groups, describe the direction of the effect. | Results - Meta-analysis |
|  | 20c | Present results of all investigations of possible causes of heterogeneity among study results. | Results - Meta-analysis (Subgroup analyses) |
|  | 20d | Present results of all sensitivity analyses conducted to assess the robustness of the synthesized results. | Results - Meta-analysis (Sensitivity analyses) |
| Reporting biases | 21 | Present assessments of risk of bias due to missing results (arising from reporting biases) for each synthesis assessed. | Results - Risk of Bias and Quality Evidence Assessment |
| Certainty of evidence | 22 | Present assessments of certainty (or confidence) in the body of evidence for each outcome assessed. | Results - GRADE assessment |
| **DISCUSSION** | | |  |
| Discussion | 23a | Provide a general interpretation of the results in the context of other evidence. | Discussion - Paragraphs 1-10 |
|  | 23b | Discuss any limitations of the evidence included in the review. | Discussion - Paragraphs 12-13 |
|  | 23c | Discuss any limitations of the review processes used. | Discussion - Paragraph 12 |
|  | 23d | Discuss implications of the results for practice, policy, and future research. | Discussion - Paragraph 13  Conclusion |
| **OTHER INFORMATION** | | |  |
| Registration and protocol | 24a | Provide registration information for the review, including register name and registration number, or state that the review was not registered. | Methods - Literature Search (PROSPERO registration) |
|  | 24b | Indicate where the review protocol can be accessed, or state that a protocol was not prepared. | Methods - Literature Search |
|  | 24c | Describe and explain any amendments to information provided at registration or in the protocol. | Not applicable |
| Support | 25 | Describe sources of financial or non-financial support for the review, and the role of the funders or sponsors in the review. | Funding section |
| Competing interests | 26 | Declare any competing interests of review authors. | Declaration of Competing Interests |
| Availability of data, code and other materials | 27 | Report which of the following are publicly available and where they can be found: template data collection forms; data extracted from included studies; data used for all analyses; analytic code; any other materials used in the review. | Not reported |

*From:*  Page MJ, McKenzie JE, Bossuyt PM, Boutron I, Hoffmann TC, Mulrow CD, et al. The PRISMA 2020 statement: an updated guideline for reporting systematic reviews. BMJ 2021;372:n71. doi: 10.1136/bmj.n71. This work is licensed under CC BY 4.0. To view a copy of this license, visit <https://creativecommons.org/licenses/by/4.0/>

**Table S2.** PRISMA2020 for Abstracts Checklist.

| **Section and Topic** | **Item #** | **Checklist item** | **Reported (Yes/No)** |
| --- | --- | --- | --- |
| **TITLE** | | |  |
| Title | 1 | Identify the report as a systematic review. | Yes |
| **BACKGROUND** | | |  |
| Objectives | 2 | Provide an explicit statement of the main objective(s) or question(s) the review addresses. | Yes |
| **METHODS** | | |  |
| Eligibility criteria | 3 | Specify the inclusion and exclusion criteria for the review. | Yes |
| Information sources | 4 | Specify the information sources (e.g. databases, registers) used to identify studies and the date when each was last searched. | Yes |
| Risk of bias | 5 | Specify the methods used to assess risk of bias in the included studies. | Yes |
| Synthesis of results | 6 | Specify the methods used to present and synthesise results. | Yes |
| **RESULTS** | | |  |
| Included studies | 7 | Give the total number of included studies and participants and summarise relevant characteristics of studies. | Yes |
| Synthesis of results | 8 | Present results for main outcomes, preferably indicating the number of included studies and participants for each. If meta-analysis was done, report the summary estimate and confidence/credible interval. If comparing groups, indicate the direction of the effect (i.e. which group is favoured). | Yes |
| **DISCUSSION** | | |  |
| Limitations of evidence | 9 | Provide a brief summary of the limitations of the evidence included in the review (e.g. study risk of bias, inconsistency and imprecision). | Yes |
| Interpretation | 10 | Provide a general interpretation of the results and important implications. | Yes |
| **OTHER** | | |  |
| Funding | 11 | Specify the primary source of funding for the review. | Yes |
| Registration | 12 | Provide the register name and registration number. | Yes |

*From:*  Page MJ, McKenzie JE, Bossuyt PM, Boutron I, Hoffmann TC, Mulrow CD, et al. The PRISMA 2020 statement: an updated guideline for reporting systematic reviews. BMJ 2021;372:n71. doi: 10.1136/bmj.n71. This work is licensed under CC BY 4.0. To view a copy of this license, visit <https://creativecommons.org/licenses/by/4.0/>

**Table S3.** Review-specific operational criteria for ROBINS-I-V2 judgments in non-randomized studies.

| **Domain** | **Low risk of bias** | **Moderate risk of bias** | **Serious risk of bias** | **Critical risk of bias** | **Review-specific considerations for this review** |
| --- | --- | --- | --- | --- | --- |
| 1) Bias due to confounding | Most key prognostic factors were appropriately measured and addressed by design and/or multivariable analysis. | Some important confounders were considered, but control was incomplete, leaving the possibility of residual confounding. | Important confounders were not adequately controlled for, or analyses relied mainly on crude comparisons without appropriate statistical adjustment. | Confounding was so severe that the observed association could not be meaningfully interpreted. | Particular attention was paid to preoperative spontaneous pain and/or percussion pain, pulp vitality/non-vital status, periapical radiolucency, retreatment status, and number of treatment visits. Additional factors such as tooth type/location, systemic disease, operator-related factors, and treatment protocol variability were considered when relevant. |
| 2) Bias in selection of participants | Participants were selected using clearly defined and appropriate eligibility criteria, and the sample appeared representative of the target clinical population (e.g., consecutive or routine clinical cases). | Minor concerns were present regarding sampling or representativeness, but major distortion of the exposure–outcome relationship was unlikely. | The participant selection process was insufficiently described or likely related to both exposure status and flare-up risk. | Selection processes were clearly related to both exposure and outcome, making the comparison seriously biased. | Lower risk was assigned when studies appeared to include consecutive or clearly defined clinical cohorts. Higher risk was assigned when exclusions, referral patterns, or unclear case selection could have influenced both exposure classification and flare-up ascertainment. |
| 3) Bias in classification of exposures | Exposure status was clearly defined and classified using objective clinical, radiographic, or treatment-record information. | Exposure classification was generally appropriate, but some ambiguity or likely non-differential misclassification remained. | Exposure definitions were unclear, inconsistently applied, or prone to substantial misclassification. | Exposure classification was fundamentally unreliable or incompatible with the intended comparison. | Typical exposures included sex, age, tooth position, tooth type, number of visits, retreatment status, pulp vitality, periapical lesion status, instrumentation type, and preoperative symptoms or medication use. Record- or chart-based definitions were judged more favorably than vague or incompletely described classifications. |
| 4) Bias due to deviations from intended exposures/interventions | Deviations or co-interventions were limited, balanced across exposure groups, or unlikely to materially influence flare-up occurrence. | Some differences in co-interventions or treatment delivery may have existed, but a major influence on the observed association was unlikely. | Important co-interventions or protocol differences likely varied across exposure groups and could have materially influenced flare-up occurrence. | Deviations or co-interventions were so strongly linked to exposure that the target association could not be separated from them. | In this review, many exposures reflected recorded clinical characteristics or treatment conditions rather than investigator-assigned interventions. Accordingly, this domain was generally judged at low risk unless a substantial imbalance in operator-related factors or treatment protocols was evident. |
| 5) Bias due to missing data | Exposure and outcome data were essentially complete, or missingness was minimal and unlikely to be related to both exposure and flare-up occurrence. | Some concern existed because missingness, follow-up completeness, or emergency-visit capture was not fully described, although major bias was not clear. | Missing data were substantial, poorly described, or likely related to exposure and/or flare-up occurrence. | Missing data was extensive and likely caused major distortion of the results. | Lower risk was assigned when studies provided explicit evidence of complete or near-complete ascertainment. Moderate risk was commonly assigned when missingness or follow-up completeness was not clearly described, even if major bias could not be confirmed. |
| 6) Bias in outcome measurement | Flare-up was defined and measured in a clinically specific and reasonably consistent manner, typically as severe pain and/or swelling requiring an unscheduled visit and additional treatment within a clearly defined postoperative period. | Minor differences in ascertainment window or outcome documentation were present, but outcome assessment remained broadly compatible with the review definition. | Outcome definitions were vague, broader than the target concept, or inconsistently applied across participants. | Outcome measurement was fundamentally incompatible with the review question (e.g., reflecting routine postoperative pain rather than clinically significant flare-up). | Lower risk was assigned when studies required unscheduled emergency consultation/intervention as part of the flare-up definition. Greater concern was assigned when definitions were looser, ascertainment windows were unclear, or chart-based capture of emergency events appeared incomplete. |
| 7) Bias in selection of the reported result | Sufficient data were available for the planned syntheses, and reporting did not appear to depend on statistical significance. | Some reporting limitations were present, but major selective reporting was not evident. | Selective reporting was suspected because only significant findings, partial analyses, or insufficient data for reconstruction were presented. | Reporting was so selective that the study could not contribute to a meaningful estimate. | Lower risk was assigned when studies reported extractable 2 × 2 data or sufficient effect estimates for the target comparisons. Higher risk was assigned when only selected significant predictors were emphasized or when non-significant results could not be adequately reconstructed. |

**Table S4.** Study characteristics of the included articles.

| Author (Year) | Country | Study Design | n | Flare-Up Definition | Risk Factors Evaluated | Flare-up rate (%) | Statistical analysis methods used | Risk factors considered significant |
| --- | --- | --- | --- | --- | --- | --- | --- | --- |
| Nosrat et al. (2023) | USA | Retrospective observational study | 3,666 | Cases requiring an unexpected emergency visit due to pain within 14 days of treatment | sex, age, systemic disease, preoperative pain (questionnaire), preoperative percussion pain, tenderness, tooth type, tooth position, preoperative CBCT, non-vital tooth, periapical lesion, multiple visits, preoperative antibiotics | 3.95% | Multivariate logistic regression analysis | sex, tooth type, systemic disease, preoperative pain, percussion pain, tenderness, periapical lesion, multiple visits, preoperative antibiotics |
| Magar et al. (2022) | Saudi Arabia | Retrospective observational study | 1,000 | Pain or swelling within 1-3 days after root canal treatment, requiring emergency response | sex, age, tooth type, number of root canals, re-treatment, systemic disease, multiple visits, non-vital tooth, periapical lesion | 9.40% | Fisher's exact test, chi-square test | tooth type, number of root canals, systemic disease, non-vital tooth, periapical lesion |
| Makanjuola et al. (2018) | Nigeria | Randomized controlled trials (RCTs) | 120 | Cases of severe pain or swelling within 7 days of treatment that resulted in an unscheduled visit and treatment | Rotary NiTi-file vs. Hand SS-file | 5.83% | Chi-square test, independent t-test | no significant difference (P=0.243) |
| Azim et al. (2017) | USA | Retrospective observational study | 951 | Acute pain and swelling that occur within 3 days of treatment and require emergency response | age, sex, tooth position, tooth type, systemic disease, non-vital tooth, re-treatment | 2.31% | Logistic regression, chi-square test | non-vital tooth, re-treatment, age (over 50) |
| Nair et al. (2017) | India | Retrospective observational study | 1,725 | If pain or swelling occurs within 3 days of treatment and requires emergency medical attention | age, sex, tooth position, tooth type, number of root canals, re-treatment, multiple visits, non-vital tooth | 1.97% | Pearson's chi-square test, Fisher's exact test | age (40-60), sex, pulp diagnosis |
| Onay et al. (2015) | Turkey | Retrospective observational study | 1,819 | Pain or swelling that occurs after treatment requires urgent consultation and aggressive treatment | age, sex, tooth position, tooth type, non-vital tooth, number of root canals, re-treatment, multiple visits, type of root canal irrigation | 3.24% | Chi-square test, Fisher's exact test, logistic regression | non-vital tooth, multiple visits |
| Pamboo et al. (2014) | India | Retrospective observational study | 1,023 | Severe pain or swelling within 1 week of treatment requires an emergency visit for treatment | age, sex, tooth position, tooth type, periapical lesion, no preoperative medication, multiple visits, used files | 2.35% | Chi-square test, logistic regression | sex, periapical lesion, no preoperative drug use, hand file |
| Tanalp et al. (2013) | Turkey | Retrospective observational study | 382 | Severe pain or swelling within 3 days of treatment requires urgent medical attention | age, sex, non-vital tooth, preoperative pain (spontaneous pain), periapical lesion, multiple visits | 3.40% | Chi-square test | preoperative pain, periapical lesion, multiple visits |
| Alves (2010) | Brazil | Prospective clinical study | 408 | Pain or swelling during treatment that requires an emergency visit and intervention | age, sex, tooth position, tooth type, preoperative drug use, re-treatment, multiple visits, non-vital tooth, periapical lesion, root apex patency | 1.71% | Chi-square test | periapical lesion |
| Iqbal et al. (2009) | USA | Retrospective observational study | 6,580 | Post-treatment pain or swelling that requires unscheduled consultation and emergency treatment | tooth type, periapical lesion, preoperative pain (history of pain), re-treatment, multiple visits, root canal filling method, and used files | 0.39% | Chi-square test, logistic regression | periapical lesion, multiple visits |
| Chen et al. (2007) | Taiwan | Prospective observational study | 658 | Pain and swelling requiring an emergency visit and urgent treatment within 3 days of treatment | age, sex, tooth position, tooth type, periapical lesion, preoperative pain (spontaneous pain), preoperative percussion pain, non-vital tooth, re-treatment, quality of root canal filling, surgeon's experience | 1.98% | Chi-square test | preoperative pain (spontaneous pain), preoperative percussion pain, root canal overfilling |
| Cheng et al. (2006) | China | Retrospective observational study | 3,696 | Pain or swelling that occurs after treatment requires emergency consultation and aggressive treatment | age, sex, systemic disease, tooth position, tooth type, preoperative pain (questionnaire), preoperative percussion pain, sinus tracts, non-vital tooth, types of intracanal medicaments, periapical lesion, surgeon's experience | 4.98% | Chi-square test, logistic regression | age (over 20), tooth position, preoperative percussion pain, non-vital tooth |
| Imura & Zuolo (1995) | Brazil | Prospective observational study | 1,012 | Pain and swelling requiring urgent visits and intervention after treatment | age, sex, tooth position, tooth type, non-vital tooth, multiple visits, re-treatment, preoperative pain (spontaneous pain), periapical lesion, preoperative medication | 1.58% | Chi-square test | preoperative pain, periapical lesion, multiple visits, re-treatment, preoperative analgesic use |
| Mor et al. (1992) | USA | Retrospective observational study | 334 | Pain and swelling requiring unscheduled visits and treatment | sex, age, tooth type, non-vital tooth, periapical lesion, preoperative antibiotics, multiple visits | 4.19% | Chi-square test | non-vital tooth |
| Walton & Fouad (1992) | USA | Prospective observational study | 946 | Pain or swelling that occurs within days of treatment and requires an emergency visit and intervention | age, sex, tooth position, tooth type, sinus tracts, multiple visits, preoperative antibiotics, preoperative analgesics, re-treatment | 3.17% | Chi-square test | patients with severe symptoms, periapical lesion with painful pulp necrosis, preoperative analgesic use |

**Table S5.** Univariable meta-regression analyses exploring study-level effect modifiers of pooled associations with endodontic flare-ups.

| Outcome | Covariate (study-level, proportion 0-1) | β | SE | t | p | p* | k | τ²_resid |
| --- | --- | --- | --- | --- | --- | --- | --- | --- |
| Age | Female proportion | -0.39 | 2.04 | -0.19 | 0.853 |  | 11 | 0.29 |
|  | Mandible proportion | -3.78 | 3.43 | -1.1 | 0.307 |  | 9 | 0.338 |
|  | Multiple-visit proportion | 2.62 | 1.66 | 1.57 | 0.177 |  | 7 | 0.211 |
|  | Non-vital teeth proportion | -1.44 | 2.02 | -0.71 | 0.497 |  | 10 | 0.33 |
|  | Apical radiolucency proportion | -4.89 | 1.76 | -2.78 | 0.032 | * | 8 | 0.175 |
|  | Re-treatment proportion | -1.56 | 1.7 | -0.92 | 0.394 |  | 8 | 0.439 |
| Sex | Age ≥50 proportion | 0.62 | 1.43 | 0.43 | 0.674 |  | 11 | 0.155 |
|  | Mandible proportion | -0.51 | 2.68 | -0.19 | 0.854 |  | 11 | 0.147 |
|  | Multiple-visit proportion | 1.17 | 0.87 | 1.35 | 0.226 |  | 8 | 0.092 |
|  | Non-vital teeth proportion | 1.59 | 1.48 | 1.08 | 0.309 |  | 11 | 0.175 |
|  | Apical radiolucency proportion | -0.15 | 0.59 | -0.26 | 0.803 |  | 10 | 0.106 |
|  | Re-treatment proportion | 0.04 | 0.91 | 0.04 | 0.969 |  | 8 | 0 |
| Mandibular | Age ≥50 proportion | -1.26 | 1.69 | -0.74 | 0.481 |  | 9 | 0 |
|  | Female proportion | -0.33 | 0.82 | -0.41 | 0.693 |  | 11 | 0 |
|  | Multiple-visit proportion | -0.46 | 0.5 | -0.91 | 0.403 |  | 7 | 0 |
|  | Non-vital teeth proportion | 1.69 | 1.01 | 1.67 | 0.139 |  | 9 | 0 |
|  | Apical radiolucency proportion | 0.37 | 0.29 | 1.27 | 0.253 |  | 8 | 0 |
|  | Re-treatment proportion | 0.83 | 1.07 | 0.77 | 0.474 |  | 7 | 0 |
| Non-vital teeth | Age ≥50 proportion | -0.36 | 0.93 | -0.39 | 0.706 |  | 10 | 0 |
|  | Female proportion | 0.23 | 0.93 | 0.25 | 0.812 |  | 11 | 0 |
|  | Mandible proportion | -0.59 | 2.18 | -0.27 | 0.795 |  | 9 | 0 |
|  | Multiple-visit proportion | -0.97 | 1.83 | -0.53 | 0.626 |  | 6 | 0 |
|  | Apical radiolucency proportion | -0.36 | 1.66 | -0.22 | 0.836 |  | 9 | 0 |
|  | Re-treatment proportion | -0.68 | 1.08 | -0.63 | 0.559 |  | 7 | 0 |
| Apical radiolucency | Age ≥50 proportion | 0.58 | 0.77 | 0.76 | 0.477 |  | 8 | 0 |
|  | Female proportion | 0.91 | 0.9 | 1.01 | 0.342 |  | 10 | 0 |
|  | Mandible proportion | 1.31 | 1.75 | 0.75 | 0.482 |  | 8 | 0 |
|  | Multiple-visit proportion | -1.43 | 1.18 | -1.21 | 0.282 |  | 7 | 0 |
|  | Non-vital teeth proportion | -0.15 | 0.98 | -0.15 | 0.885 |  | 9 | 0 |
|  | Re-treatment proportion | 0.62 | 0.93 | 0.67 | 0.539 |  | 6 | 0 |
| Tooth type  (Anterior vs others) | Age ≥50 proportion | -0.24 | 0.93 | -0.26 | 0.804 |  | 9 | 0 |
|  | Female proportion | -0.99 | 1.37 | -0.72 | 0.49 |  | 11 | 0 |
|  | Mandible proportion | 1.23 | 2.21 | 0.55 | 0.595 |  | 10 | 0 |
|  | Multiple-visit proportion | 1 | 0.73 | 1.38 | 0.217 |  | 8 | 0 |
|  | Non-vital teeth proportion | 0.34 | 1 | 0.34 | 0.743 |  | 9 | 0 |
|  | Apical radiolucency proportion | -0.83 | 0.5 | -1.66 | 0.142 |  | 9 | 0 |
|  | Re-treatment proportion | 0.11 | 0.87 | 0.12 | 0.906 |  | 9 | 0 |
| Tooth type  (Premolar vs others) | Age ≥50 proportion | 1.12 | 0.94 | 1.19 | 0.273 |  | 9 | 0.065 |
|  | Female proportion | 1.37 | 1.42 | 0.97 | 0.358 |  | 11 | 0.03 |
|  | Mandible proportion | -0.63 | 2.69 | -0.23 | 0.822 |  | 10 | 0 |
|  | Multiple-visit proportion | -0.13 | 0.64 | -0.2 | 0.849 |  | 8 | 0 |
|  | Non-vital teeth proportion | -1.01 | 1.04 | -0.97 | 0.362 |  | 9 | 0.017 |
|  | Apical radiolucency proportion | 0.42 | 0.42 | 1 | 0.349 |  | 9 | 0 |
|  | Re-treatment proportion | 0.63 | 1.12 | 0.56 | 0.594 |  | 9 | 0.059 |
| Tooth type  (Molar vs others) | Age ≥50 proportion | -1.06 | 0.88 | -1.21 | 0.265 |  | 9 | 0 |
|  | Female proportion | -0.34 | 1.26 | -0.27 | 0.792 |  | 11 | 0 |
|  | Mandible proportion | -1.65 | 2.3 | -0.71 | 0.495 |  | 10 | 0 |
|  | Multiple-visit proportion | -0.43 | 0.53 | -0.81 | 0.451 |  | 8 | 0 |
|  | Non-vital teeth proportion | 0.73 | 0.92 | 0.8 | 0.451 |  | 9 | 0 |
|  | Apical radiolucency proportion | 0.01 | 0.37 | 0.02 | 0.981 |  | 9 | 0 |
|  | Re-treatment proportion | -0.74 | 0.84 | -0.87 | 0.412 |  | 9 | 0.018 |

β is on the log(RR) scale. Study-level covariates were entered as proportions (0–1). p-values are from t-tests (df = k − 2). Analyses are exploratory and hypothesis-generating.

**Table S6.** GRADE assessment of the certainty of evidence for additional risk factors.

| **Certainty assessment** | | | | | | | **Certainty** | **Importance** |  |
| --- | --- | --- | --- | --- | --- | --- | --- | --- | --- |
| **№ of studies** | **Study design** | **Risk of bias** | **Inconsistency** | **Indirectness** | **Imprecision** | **Other considerations** |  |  |  |
|  |  |  |  |  |  |  |  |  |  |
| **Relationship between age (≥50 years) and flare-ups** | | | | | | | | |  |
| 11 | non-randomised studies | serious^a^ | serious^b^ | not serious | serious^c^ | none | ⨁◯◯◯  Very low^a,b,c^ | NOT IMPORTANT |  |
|  |  |  |  |  |  |  |  |  |  |
| **Relationship between the number of root canals (multiple) and flare-ups** | | | | | | | | |  |
| 3 | non-randomised studies | serious^a^ | not serious | not serious | serious^c^ | none | ⨁⨁◯◯  Low^a,c^ | NOT IMPORTANT |  |
|  |  |  |  |  |  |  |  |  |  |
| **Relationship between the tool used (SS Hand) and flare-ups** | | | | | | | | |  |
| 3 | non-randomised studies | serious^a^ | not serious | not serious | serious^c^ | none | ⨁⨁◯◯  Low^a,c^ | NOT IMPORTANT |  |
|  |  |  |  |  |  |  |  |  |  |
| **Relationship between tooth type (incisor) and flare-ups** | | | | | | | | |  |
| 12 | non-randomised studies | serious^a^ | not serious | not serious | not serious^c^ | none | ⨁⨁⨁◯  Moderate^a,c^ | IMPORTANT |  |
|  |  |  |  |  |  |  |  |  |  |
| **Relationship between tooth type (premolar) and flare-ups** | | | | | | | | |  |
| 12 | non-randomised studies | serious^a^ | not serious | not serious | not serious^c^ | none | ⨁⨁⨁◯  Moderate^a,c^ | IMPORTANT |  |
|  |  |  |  |  |  |  |  |  |  |
| **Relationship between tooth type (molar) and flare-ups** | | | | | | | | |  |
| 12 | non-randomised studies | serious^a^ | not serious | not serious | not serious^c^ | none | ⨁⨁⨁◯  Moderate^a,c^ | IMPORTANT |  |
|  |  |  |  |  |  |  |  |  |  |
| **Relationship between analgesic and flare-ups** | | | | | | | | |  |
| 3 | non-randomised studies | serious^a^ | serious^d^ | not serious | serious^c^ | none | ⨁◯◯◯  Very low^a,c,d^ | NOT IMPORTANT |  |
|  |  |  |  |  |  |  |  |  |  |

**Explanations**

a. In the ROBINS-I-v2 evaluation, many studies are rated as "moderate" or "serious".

b. I² = 70.5%, Q = 33.93 (df = 10, p = 0.0002).

c. CIs are not statistically significant, and the direction of the effects is not consistent.

d. I² = 84.9%, Q = 13.21 (df = 2, p = 0.0014).
